# Compassion Fatigue among Healthcare, Emergency and Community Service Workers: A Systematic Review

**DOI:** 10.3390/ijerph13060618

**Published:** 2016-06-22

**Authors:** Fiona Cocker, Nerida Joss

**Affiliations:** 1 School of Public Health and Preventive Medicine, Monash Centre for Occupational and Environmental Health (MonCOEH), Monash University, Prahran 3004, Australia; 2 Melbourne School of Population and Global Health, University of Melbourne, Melbourne 3010, Australia; nerida.joss@unimelb.edu.au

**Keywords:** compassion fatigue, secondary trauma, interventions, risk factors, health, emergency, community service workers

## Abstract

Compassion fatigue (CF) is stress resulting from exposure to a traumatized individual. CF has been described as the convergence of secondary traumatic stress (STS) and cumulative burnout (BO), a state of physical and mental exhaustion caused by a depleted ability to cope with one’s everyday environment. Professionals regularly exposed to the traumatic experiences of the people they service, such as healthcare, emergency and community service workers, are particularly susceptible to developing CF. This can impact standards of patient care, relationships with colleagues, or lead to more serious mental health conditions such as posttraumatic stress disorder (PTSD), anxiety or depression. A systematic review of the effectiveness of interventions to reduce CF in healthcare, emergency and community service workers was conducted. Thirteen relevant studies were identified, the majority of which were conducted on nurses (*n* = 10). Three included studies focused on community service workers (social workers, disability sector workers), while no studies targeting emergency service workers were identified. Seven studies reported a significant difference post-intervention in BO (*n* = 4) or STS (*n* = 3). This review revealed that evidence of the effectiveness of CF interventions in at-risk health and social care professions is relatively recent. Therefore, we recommend more research to determine how best to protect vulnerable workers at work to prevent not only CF, but also the health and economic consequences related to the ensuing, and more disabling, physical and mental health outcomes.

## 1. Introduction

The compassion and empathy shown by healthcare, emergency and community service professionals can prove psychically, mentally and economically costly. In short, exposure to patients or clients experiencing trauma or distress can negatively impact professional’s mental and physical health, safety and wellbeing, as well as that of their families, the people they care for, and their employing organizations. The term compassion fatigue (CF) was coined to described the phenomenon of stress resulting from exposure to a traumatized individual rather than from exposure to the trauma itself [1]. An often extreme state of tension and preoccupation with the emotional pain and/or physical distress of those being helped can create a secondary traumatic stress (STS) for the caregiver [2,3], and, when converged with cumulative burnout (BO), a state of physical and mental exhaustion caused by a depleted ability to cope with one’s everyday environment [4,5,6], CF results.

CF is characterized by exhaustion, anger and irritability, negative coping behaviours including alcohol and drug abuse, reduced ability to feel sympathy and empathy, a diminished sense of enjoyment or satisfaction with work, increased absenteeism, and an impaired ability to make decisions and care for patients and/or clients [7]. The negative effects of providing care are aggravated by the severity of the traumatic material to which the caregiver is exposed, such as direct contact with victims, particularly when the exposure is of a graphic nature. This places certain occupations, such as healthcare, emergency and community service workers, at an increased risk of developing CF and potentially more debilitating conditions such as depression and anxiety [8], and even posttraumatic stress disorder (PTSD) [9]. These conditions are known to increase sickness absence, psychological injury claims, and job turnover, and negatively impact productivity.

**Figure 1:**
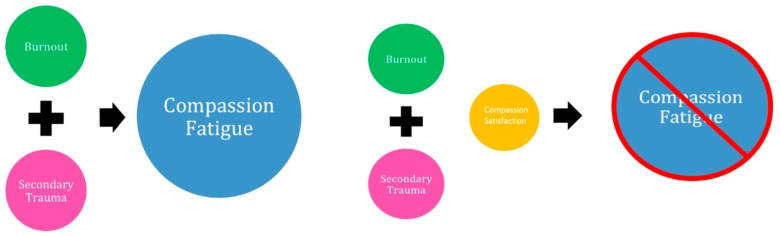
Compassion fatigue: conceptual model adapted from Middleton [28].

Compassion fatigue (CF) has been variously defined, and the related concepts of BO, STS and vicarious traumatisation (VT) are often used interchangeably and incorrectly to describe the phenomenon. BO and STS are related to CF, but as defined by Stamm, they are two distinct outcomes of exposure [10]. As demonstrated by Figure 1, BO and STS arise from separate failed survival strategies [11]. BO arises from a assertiveness-goal achievement response and occurs when an individual cannot achieve his or her goals and results in “frustration, a sense of loss of control, increased willful efforts, and diminishing morale” [11]. Alternatively, STS arises from a rescue-caretaking response and occurs when an individual cannot rescue or save someone from harm and results in guilt and distress [11]. Subsequently, STS and BO lead to CF if the aforementioned symptoms are not mediated by a third, equally important concept of compassion satisfaction (CS). CF and CS can be seen as the positive and negative consequences of working with individuals who have experienced or are currently experiencing trauma or suffering [10]. As a result, a substantial amount of evidence suggests CS is an important part of the whole [12], thus increasing the significance of building resiliency and the transformation from negative to positive aspects [10,13,14].

The most commonly used definition of CF was developed by Figley [1] and describes the concept as “a state of exhaustion and dysfunction biologically, psychologically, and socially as a result of prolonged exposure to compassion stress and all it invokes” (p. 253). This definition more effectively encompasses the multiple dimensions of CF. Lynch and Lobo [15] defined the attributes of CF as an established relationship between the caregiver and the patient/client all associated with the caregiving role and the psychological and physical responses it arouses [15]. Yoder *et al*. [16] identified triggering events based on nurses taking care of patients who were experiencing serious life-threatening situations and cases involving futile or palliative care. Lynch and Lobo [15] also identified system issues or organisational factors, such as physical and emotionally demanding assignments and extra work days as risk factors for CF, while Najjar *et al*. identified that empathy is vital to the development of CF, as the caregiver must have the ability to perceive and understand what their patient/client is experiencing and be able to communicate this understanding [17]. Finally, the psychological response to the conflicting elements of empathy and suffering provides the foundation for ongoing stress and subsequent CF [15]. In summary, CF results from “the change in empathetic ability of the caregiver in reaction to the prolonged and overwhelming stress of caregiving” [15].

CF is most commonly measured using the validated Professional Quality of Life (ProQoL) scale. The overall concept of ProQoL is a complex milieu of characteristics of the work environment (organization, tasks), the individual’s personal characteristics, and their exposure to primary and secondary trauma in the work setting. In short, ProQoL refers to the quality one feels in relation to their work as a caregiver, and both the positive and negative aspects of doing one’s job. As such, the ProQoL scale measures both pre-cursors of CF (BO and STS), and CS.

Since the mid-1990s [1,18,19,20] the emotional, cognitive, and physical consequences of providing professional services to trauma victims and survivors have been addressed in the literature and several conceptual models have been developed in attempt to explain them. However, the majority of research to date has focused on identifying the prevalence and predictors of CF in a unique occupational group such as nurses [21,22], therapists [23,24], community service workers, and healthcare professionals in hospital emergency departments or intensive care units [25]. While these studies have gone some way to illuminate how CF can be addressed, their findings cannot be generalised to working populations beyond the healthcare sector. Furthermore, despite the identification of modifiable individual and organisational risk factors used to inform the development of interventions and programs to prevent and treat CF, few studies have attempted to examine and interrogate the quality of these preventive measures. These include workload intensity, inadequate rest time periods between shifts, task repetitiveness [3], low control and low job satisfaction [26], poor resilience, lack of meaningful recognition, and poor managerial support [27]. As a result, employers and managers in the healthcare, emergency and community services sectors have very little conclusive evidence as to the best way to prevent CF, and physical and mental health outcomes, when its known risk factors—exposure to traumatised patients and clients—are inherent in the type of work that the employees supervise and undertake.

The ultimate goal of CF research is to create healthy caregivers that are able to master the art of resiliency and return quickly to high-functioning behaviours, both at work and outside the workplace, after exposure to the traumatic experience of a patient or client. As experiencing secondary trauma could be considered an inherent risk for the occupations of interest in this paper, the actual job exposures may be difficult to modify. Therefore, interventions that promote individual resilience and educate at-risk workers about effective coping strategies in response to these adverse job exposures are equally important and likely to have significant health and economic benefits, as they reduce not only STS, BO and CF, but also the risk of more serious mental health disorders such as anxiety and depression, the quality of life and productivity consequences of which are well documented. Therefore, this systematic review aimed to: (i) identify existing evidence on interventions to reduce CF in healthcare, and emergency and community service professionals; and (ii) determine the most effective workplace-based strategies for reducing CF, directly or via modifying its recognised individual and organisational risk factors and/or promoting Compassion Satisfaction.

## 2. Methods

### 2.1. Search Strategy

The following search strategy was carried out using the major relevant database search engines (*i.e*., EMBASE, CINHAL, PsychInfo, Web of Science, PubMed, and Scopus). Search terms were divided into four groups. Group 1 related to CF, related concepts, and variants or derivatives such as VT or STS. Group 2 included at-risk occupation types commonly exposed to known risk factors for CF such as frontline health, emergency and community service workers. Group 3 included terms related to the study design, for which we used the Cochrane search terms for identifying interventions and trials (e.g., intervention or training or program or efficacy or randomized control group). Group 4 related to employment type or derivatives of related words such as employees, workers and professionals.

The search was limited to peer-reviewed articles published in English between January 1990 and December 2015. Both authors (Fiona Cocker and Nerida Joss) screened article titles and abstracts to determine eligibility. In addition, a hand search of key journals and reference lists of all studies selected for inclusion in the analysis was conducted. Appendix Table A1 contains a summary of the search strategy.

### 2.2. Inclusion and Exclusion Criteria

The inclusion criteria dictated that the studies contained a quantitative evaluation of an intervention that reported outcomes on a standardized and validated measure for compassion fatigue. This measure could be any of the validated versions of the most commonly used Professional Quality of Life (Pro-QoL) or a less common, but equally valid measure of CF. Alternatively, the outcomes could be one of the sub-scales of the Pro-QoL; CS, a protective factor, or STS or BO, both of which are known risk factors for CF. Finally, the outcome could be a subscale of a general health measure that has evidence of validity as a CF, BO, STS, or CS screening tool (e.g., Maslach Burnout Scale [29], or The Resilience Scale [30]). The intervention had to target CF directly or indirectly through a known risk factor for CF, such as those reviewed in the introduction (lack of meaningful recognition, years of experience, higher job satisfaction, and poor psychosocial work climate). The intervention settings could be in work or non-work settings. Studies were excluded if they did not report on CF, focused on prevalence of CF only, or used a qualitative methodology.

### 2.3. Data Extraction

The variables extracted covered intervention descriptors, sample characteristics, implementation characteristics, quality of the research design (use of control group, random allocation), and outcome indicators. We did not use a quality rating score in the analysis, as it introduces subjectivity and is prone to incomplete data. Coding instructions and guidelines were developed by the first and second authors in order to reduce the subjectivity of decisions made. On completion of the coding, the first author (Fiona Cocker) independently checked the coding of each of the papers. Instances in which disagreement with the initial coding decisions occurred were resolved by consensus between both authors (Fiona Cocker and Nerida Joss).

### 2.4. Statistical Analysis

Our primary outcomes were the components of the ProQoL: BO, STS and CS. Due to the small number of studies identified (*n* = 13), no statistical analysis was undertaken, and meta-analysis was not possible.

## 3. Results

**Figure 2:**
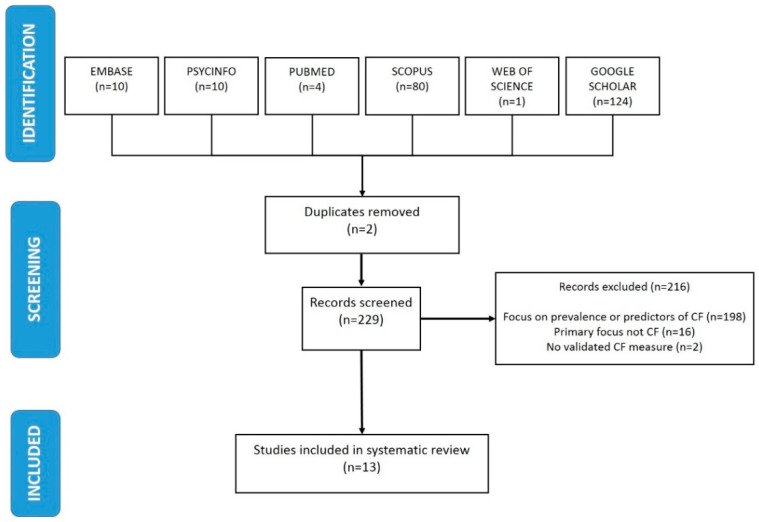
Flow diagram of study selection. CF=Compassion Fatigue.

Figure 2 presents the PRISMA (Preferred Reporting Items for Systematic Reviews and Meta-Analyses) flow diagram summarizing the inclusion and exclusion decisions made by both authors (Fiona Cocker and Nerida Joss). Due to the small number of hits generated (229), all articles were carefully inspected to determine whether they met criteria for inclusion, as opposed to making exclusion decisions solely on the basis of the initial title screening. Following this review, two duplicate articles were removed, and 216 articles were excluded. The majority of the studies (*n* = 198, 91.6%) were excluded, as they focused solely on prevalence of CF, or a CF risk factor, within a particular occupational group. The remainder of articles were excluded, as they did not evaluate the effectiveness of a CF prevention or intervention program, their primary focus was not CF, or they did not have a specific, validated measure of CF or factors which contribute to CF. For example, studies were excluded if they had a PTSD or stress-related outcome measure, measures of general psychosocial functioning, or well-being scales that have not been established in the literature as valid indicators of CF. The application of these inclusion criteria resulted in a total of 13 studies being deemed suitable for the detailed, systematic review and data extraction.

### 3.1. Characteristics of Included Studies

Characteristics of included studies (*n* = 13) are presented in Table 1. Of the included studies *n* = 11 (84.6%) were conducted in the USA [31,32,33,34,35,36,37,38,39,40,41], one in Australia [42], and one in Israel [43]. Ten of the thirteen studies included nurses (76.9%), with three studies specifically focusing on oncology nurses [32,34,35], one on pediatric nurses [43], and one on emergency nurses [39]. Other occupational groups represented were social workers (*n* = 2, 15.4%) [31,33], chaplains (*n* = 1, 7.7%) [33], hospice workers (*n* = 1, 7.7%) [40], disability sector workers (*n* = 1, 7.7%) [42], and miscellaneous medical staff (*n* = 3, 23.1%) [37,38,41]. No studies targeting emergency service workers were identified. The 13 studies included represented a total sample size of 671 (*M* = 52, *SD* = 43), with sample sizes for the individual studies ranging from seven [36] to 154 [34]. Of those that reported mean age (*n* = 7, 53.8%) [31,34,35,38,40,42,43], averages ranged from 40.5 years [35] to 49.3 years [43], and of those which reported sex distribution (*n* = 11, 84.6%) [31,32,33,34,35,36,38,39,40,42,43], the majority of subjects were female (58.8% [42]–100% [43]). All included studies had a follow-up period ranging from three weeks [31] to six months [35] post-intervention.

### 3.2. Outcome Measurements

Ten (76.9%) of the included studies used a version of the Professional Quality of Life Scale (ProQoL) [10] to measure CF, as well as its subscales CS, BO and STS. Other measures of CF used were The Compassion Fatigue Scale (CFS), both original [1] and revised [44] versions, designed to assess both secondary trauma and job burnout, and the Compassion Satisfaction and Fatigue Test (CSFT) [12]. Three (23.1%) of the included studies [31,32,39] measured only one type of outcome (e.g., ProQoL, Version 5, Manufacturer, City, Country). The remaining ten included articles [33,34,35,36,37,38,40,41,42,43] measured the following outcomes in addition to CF (Table 1): (i) burnout [34,35,38,42,45]; (ii) team building [33]; (iii) impact of traumatic events [34]; (iv) satisfaction with work or life [34,42]; (v) empathy [36]; (vi) resilience [36,41]; (vii) depression, anxiety, stress, or perceived stress [36,37,38,42]; (viii) self-efficacy [43], self-esteem [43]; mastery and hope [43]; mindfulness [37,38], healthcare consumer assessment [37], general health [42]; grief [40]; work environment [40]; and coping [41].

### 3.3. Intervention Design

All of the interventions evaluated in the included studies were individually focused, and the majority (*n* = 7, 53.8%) focused on stress reduction using yoga and/or mindfulness [31,37,42], structured meditation [32,39], music therapy [33], or a combination thereof [38]. Of the remaining four included studies, two evaluated interventions focused on building individual resilience [34,35], one aimed to build professional self-efficacy [43], and Stanton *et al*. [36] used Transcranial Magnetic Stimulation to increase resilience and empathy, and reduce stress. Eleven studies described single-faceted interventions focusing on yoga, mindfulness, meditation, or music therapy [31,32,33,37,38,40,42], resilience and coping [34,35,41], or transcranial magnetic stimulation [36]. In contrast, Berger *et al*. [43] and Flarity *et al*. [39] described more complex interventions involving on multiple, interactive sessions focused on promoting professional self-efficacy, improving theoretical knowledge, and assigning homework tasks, and individual and group exercises, guided imagery, take home materials including print-outs, DVDs and music CDs, and access to educational resources and publications, respectively. The included studies differed in their methodology; ten studies (76.9%) [31,32,33,34,35,36,37,39,41,42] used a pre/post design, two studies (15.4%) used randomised controlled designs (RCTs), one with a waitlist control group [38], the other with a no treatment, concurrent control group [40]. Finally, Berger *et al*. [43] used a quasi-random control trial with a wait list control group. Intervention periods of the included studies ranged from three to twelve weeks (*M* = 6.1, *SD* = 2.7), session length ranged from 16 to 240 min (*M* = 78.9, *SD* = 58.6), and the frequency of sessions ranged from once a week to five times a week (*M* = 1.5, *SD* = 1.2). None of the included studies assessed intervention quality using a validated measure, and the majority of studies (*n* = 10, 76.9%) used unrepresentative, convenience samples.

### 3.4. Effect of Interventions to Prevent or Manage Compassion Fatigue

The follow-up intervals ranged from three weeks [31], immediately post-intervention, to 6 months [35] after the baseline measurements. Eight (61.5%) of included studies reported a significant difference post-intervention in either CF, or one of the ProQoL subscales BO, CS, or STS. More specifically, five studies (38.5%) [32,34,35,39,41] reported significantly reduced BO and STS, risk factors for CF, and three studies (23.1%) [38,39,43] reported significantly increased CS, a protective factor in the development of CF. Flarity *et al*., the one study to achieve reduction in BO and STS and an increase is CS, conducted an intensive, two-level intervention amongst emergency nurses involving, firstly, a four hour, interactive group seminar focused the “…origin of CF, the physiological effects, signs and symptoms of CF and BO, as well as the factors associated with emergency nursing that may lead to CF and BO”, as well as providing information about how to prevent and treat CF using the five elements identified by Gentry *et al*. [44]; self-regulation, intentionality, perceptual maturation/self-validated caregiving, connection and self-care. Secondly, participants were given multimedia resources such as printed seminar handouts, a guided imagery CD, access to a website with CF, CS and resiliency educational resources and publications, and DVD which informed them of Gentry’s [44] aforementioned five elements. This is, by far, the most comprehensive intervention evaluated by the included studies and, not surprisingly, has the most significant, positive outcomes. Unlike the other twelve interventions evaluated, this intervention focuses on teaching participants: (i) about CF; (ii) how to recognise, and actively prevent and treat CF in themselves and their colleagues; and (iii) provides them with tools and resources to consolidate these learnings which is likely to increase the probability of these positive outcomes remaining long term. However, this is yet to be determined.

## 4. Discussion

This systematic review identified the evidence on interventions designed to reduce CF in health, emergency and community service workers to determine the most effective workplace based strategies for reducing CF directly or via modifying its recognised individual and organisational risk factors. Despite the significant attention given to measuring the prevalence of CF in this cohort of workers, there is a lack of information and evidence about effective interventions designed to reduce CF in these occupational groups. We have found that, despite recognition of the threat exposure to secondary trauma poses to the mental health and wellbeing of certain at-risk occupational groups, and the established existence of wellness programs to combat CF and related concepts, rigorous, academic evaluation of evidence on this topic has only recently emerged in the last few years, with the earliest study published in 2011 with a group of 80 pediatric nurses in Israel, thus indicating the relative novelty of interventional research in this area. By consolidating the small amount of evidence available we have been able to identify promising interventions in this area as well as the evidence gaps and areas in need of research attention in the future. In doing so, the subsequent evidence based workplace-based interventions have the potential to reduce CF and more serious, chronic and economically costly mental disorders, to the benefit of individual workers, employers and the broader society and economy through the retention of healthy, productive workers who service those in need of healthcare and social assistance.

### 4.1. Study Population Characteristics

The majority of studies identified in our search have been generated from the US (*n* = 11), with nurses as the occupational group of interest (*n* = 10). As such, nurses were disproportionally represented, limiting the generalizability of findings to other equally at-risk occupations such as police, fire fighters, paramedics and other health and community service workers. Further, the majority of subjects represented in the included studies were female (58.8% [42] to 100% [43]), which, while reflective of the healthcare and social assistance industry to which many of the at-risk populations belong [46], once again, reduces the level of generalizability as other occupational groups at-risk of CF, such as the male-dominated emergency services [46] are not included. Older workers were also disproportionally represented, with averages ranging from 40.5 years [35] to 49.3 years [43], thus limiting applicability of the findings to younger workers who may be at risk of developing CF. More specifically, evidence has identified that both age and years of professional experience were protective factors for STS, CF, or BO [47,48,49]. In addition to the over-representation of several demographic characteristics, some individual characteristics (e.g., educational attainment) and organisational factors (e.g., long work hours and caseloads with high percentages of trauma patients) which have been associated with an increased incidence of STS and CF [50,51], were not considered.

### 4.2. Study Design and Methodological Quality

Comparison between studies was difficult given the heterogeneity of the interventions themselves and the lower level of methodological quality for the majority of studies. The majority of studies were of low to moderate quality (*n* = 11), with only two RCT studies included in our review [38,40]. The majority of studies (*n* = 10) employed a pre-post design, most of which did not include a long-term follow up (≥8 weeks). Additionally, the length of the intervention period varied considerably from a single four hour session [39] to six, one hour sessions once a week for 12 weeks [43]. This is a concern, as significant behavioural and personal routine modification is required in some cases, without any complementary changes being made to the work-related risk factors such as reduced exposure to traumatised patients or clients or increased rest between shifts. Change is also unlikely within this short time period [52,53], thus making significant improvement in CS or a significant decrease in BO and/or STS similarly improbable. Finally, in addition to highly variable methodological design and quality, most studies included (*n* = 12) have small sample sizes with less than 100 subjects, which reduce statistical power, increase the possibility of type II error, and reduce the ability for statistical tests to detect significant differences between values.

### 4.3. Effectiveness of CF Interventions

The thirteen included studies in our search demonstrated mixed or no effects. While ten studies reported significant improvement in at least one element of CF, no study reported positive change on all three indicators (STS, BO, CS) and only one study had a follow up period of longer than eight weeks. This makes it difficult to determine with any certainty whether these effects were likely to be sustained over time.

When we considered the findings of the included studies by the content of the interventions evaluated, didactic and ecologic music therapy interventions were shown to be ineffective, as was grief resolution, which involves expression of grief feelings, connecting socially with colleagues experiencing similar feelings, and participation in a grief ritual to farewell patients who had died, Transcranial Direct Current Stimulation, and mindfulness education. In contrast, structured meditation using an audio CD [32], and interactive group seminars followed by individual and group exercises such as guided imagery, and multimedia resources (printed handouts, DVD, guided imaging/music CD, a website with CF and CS, and resiliency educational resources and publications) resulted in a significant decrease in BO. However, the most promising trend was for the effectiveness of interventions involving an element focused on teaching and/or bolstering resilience [35,39,41], all of which showed improvement in BO, and two of which demonstrated a reduction in STS and BO, and an improvement in CS. These findings are encouraging, as they suggest that workers in at-risk occupational groups can be taught to cope with the known risk factors for the development of CF, which are also, unfortunately, unavoidable parts of their job. It also suggests a need to invest in programs such as the Accelerated Program for Compassion Fatigue (ARP), developed by Gentry *et al*. [44], a five-session model for the treatment of the deleterious effects caregivers experience as a result of their care giving work [54] through the promotion of resilience and self-efficacy. Participants in the ARP not only report a reduction in CF symptoms, they also feel more empowered, more energetic, and have a stronger sense of self-worth. Designed to reduce the intensity, frequency and duration of symptoms associated with Compassion Fatigue, ARP aims to help at-risk workers identify symptoms of CF, recognise CF triggers, identify and utilize existing available resources, review personal and professional history to the present day to identified those at increased risk, master arousal reduction methods, resolve any impediments to efficacy, initiate conflict resolution, and initiate a supportive aftercare plan-in collaboration with their employer or supervisor.

The ARP program borrows from PTSD literature and focuses on the restorative quality of personal self-awareness and promotes the sharing of stories and debriefing to assist those experiencing CF in rebuilding their professional and personal life quality. The ARP also advocates the promotion of self-compassion in order to encourage individuals to challenge a negative internal dialogue and focus on shifting their automatic thoughts and beliefs to reflect more positive outlook. Finally, the program promotes the development of a combination of “Resiliency Skills” of “Antibodies” which, have been shown to be correlated with lessened CF, greater job satisfaction, better quality of life and lessened anxiety [54,55]. They are as follows: (i) self-care and revitalisation; (ii) connection and support; (iii) intentionality, or eradicating stress and shifting from reactive to intentional behavior [56]; (iv) self-regulation, which involves developing the ability to intentionally control the activity and lessen the energy of the Autonomic Nervous System while engaged in the activities of daily living. For some, this may prove as simple as relaxing the muscles while encountering the myriad of perceived threats that emerge throughout each workday; and (v) perceptual maturation, which is a cognitive skill and involves maturing the perceptions of self towards resiliency and the perceptions of the workplace, to render them less toxic.

### 4.4. Limitations

While a thorough search strategy was designed to undertake this systematic review, limitations should be considered in the interpretation of results. As with any search, despite searching six major databases, some studies may have been missed. Our search was limited to only search English language journals, and, therefore, studies published in a language other than English might have missed.

### 4.5. Implications for Future Research

This review indicates that there is some promising evidence emerging about interventions to reduce CF, in particular in nurses. However, given the small number of published studies to date, it is difficult to determine the impact on this or other occupations. The review has shown that in particular, there is a gap in research conducted in many emergency occupations such as police and fire fighters, and other health community services such child protection and disability support workers, and disability and human service workers, and further research is needed using more rigorous study designs and representative samples. These groups are particularly susceptible to developing CF due to the nature of their work. In addition, future research could focus on the impact of CF interventions in a more diverse range of at-risk occupation groups, over-sample younger aged workers and men in order to the determine the effectiveness of interventions designed to reduce CF, or prevent and manage known risk factors, in these established at-risk populations.

This can be best understood if we consider CF, or its risk and protective factors BO, STS, and CS, as upstream determinants of common mental disorders such as a depression and anxiety, the health and economic cost of which can be considerable within the employed population [57,58,59]. More specifically, by reducing the incidence of CF, future cases of depression and anxiety could be prevented, thus reducing the related health and economic consequences of these conditions. The effectiveness of this approach was demonstrated in the work of LaMontagne *et al*. [60], who estimated that the potential cost saving of eliminating job strain as an avoidable predictor of depression was $730 million AUD for one year and $11.8 billion AUD over a lifetime. However, it must be noted that, although workplace psychosocial stressors, such as job strain, have been linked to poor mental and physical health in a growing body of scientific evidence, the exploration of CF in such a relationship is a relatively newer concept. Therefore, future work is required to: (i) establish the degree to which, if any, CF increases a worker’s future risk of depression or anxiety, after accounting for other known risk factors; and (ii) establish whether CF can be effectively reduced or eliminated by a combination of work- and worker-directed interventions across occupational groups. Once established, the epidemiological and economic modelling approach used by LaMontagne *et al*. [60] could be used to highlight the financial value of investing in the mental health workers at-risk of CF in addition to the existing legal or moral motivators, to the ultimate benefit of employers, workers, and the patients and clients they serve.

Furthermore, there has been minimal effort made to apply the aforementioned findings about health, allied health, and community service workers to reduce CF and its negative health, wellbeing and safety consequences. Therefore, we recommend a systematic review be conducted to determine the prevalence of CF across occupation types to assist in identifying those most at risk, and, therefore, in most need of intervention. Finally, although it is difficult to make definitive conclusions due to the quality of the evidence in this review, the interventions that contain at least one element of resilience training appeared to have the most effect on CF. Therefore, we suggest researchers, employers and managers consider this when designing interventions to reduce CF in the future.

## 5. Conclusions

To our knowledge, this is the first review to attempt to evaluate the evidence of the effectiveness of CF interventions in at-risk health, emergency and social care professions. Results revealed that, despite an awareness of the prevalence of CF in these at-risk workers, there is a lack of information and evidence about effective workplace based strategies to reduce CF in these occupational groups via modifying its recognised individual and organisational risk factors. Therefore, we recommend more research to determine how best to protect vulnerable workers in order to prevent CF, as well as the potentially more significant health and economic consequences related to the subsequent physical and mental health outcomes.

## Figures and Tables

**Table 1:** Summary of included studies reporting on CF interventions (*n* = 13).

First Author, Year and Country	Occupational Group (N), % Female and Age	Target of Intervention ^a^	Intervention Design/Content	Measure of CF	Other Measures ^c^	Follow-Up	Effectiveness Against Targeted Outcomes	Conclusions
[31]	Social workers (*n* = 11) Intervention Group (*n* = 5) Control Group (*n* = 6) 90.9% female Age: *M* = 44.73, *SD* = 14.4	BO, CS and STS	Pre/Post—One hour yoga and mindfulness program once a week for 3 weeks.	ProQoL, V5 ^b^	None	3 weeks post baseline	**INTERVENTION GROUP MEAN (pre *vs.* post)** CS (↑) (*p* = 0.121) BO (↓) (*p* = 0.249) STS (↓) (*p* = 0.155) **CONTROL GROUP MEAN (pre *vs.* post)** CS (↓) (*p* = 0.004) BO (↓) (*p* = 0.352) STS (↓) (*p* = 0.866)	Significant decrease in CS for controls could suggest the absence of a coping resource (*i.e.*, yoga, mindfulness). Lower CS could predict BO, STS and CF.
[32]	Oncology nurses (*n* = 15) 100% female Age: not reported	CF and CS	Pre/Post—16 min of structured meditation, using an audio CD, 5 days a week for 4 weeks.	ProQoL, V5	None	4 weeks post baseline	**INTERVENTION GROUP ONLY (pre *vs*. post**) CS (↑) (*p* = 0.27) BO (↓) (*p* = 0.003) STS (↓) (*p* = 0.47)	Effective in reducing stress and cultivating self-compassion. Time poor nurses suggest commitment might be difficult.
[33]	Nurses, social workers and chaplains (*n* = 17) 64.7% female Age Range = 28–60 years	CF	Pre/Post—1 h music therapy groups delivered weekly for 6 weeks. Treatment group 1 received an ecological music therapy approach; treatment group 2 received a didactic music therapy approach.	Compassion Fatigue Scale (CFS)	TBQ	6 weeks post baseline	**INTERVENTION GROUP 1 (pre *vs*. post)** CFS (↑) (*p* = n.s.) **INTERVENTION GROUP 2 (pre *vs*. post)** CFS (↓) (*p* = n.s.)	No significant difference in CFS. Significant improvement in team building in both groups.
[34]	Oncology staff (*n* = 154) 92.7% female Age: *M* = 40.5	CF	Pre/Post—90 min sessions on CF resiliency once a week for 5 weeks and a 2 day facilitator’s course.	ProQoL R-IV	MBI Revised Impact of Events Scale Nurse Job Satisfaction Scale	3 months	**INTERVENTION GROUP 1 (pre *vs*. post)** CS (↑) (*p* ≤ n.s.) BO (↓) (*p* ≤ 0.01) STS (↓) (*p* ≤ 0.01)	Staff rated the program highly, relevant to the job. BO and CF both decreased significantly.
[35]	Oncology nurses (*n* = 13) 85.7% female Age: *M* = 43.9	CF	Pre/Post—90 min sessions on CF resiliency once a week for 5 weeks.	ProQoL IV	MBI-Human Services Survey	5 weeks, 3 months and 6 months post baseline	**INTERVENTION GROUP (pre- *vs*. post)** CS (↑) (*p* = n.s.) BO (↓) (*p* = n.s.) STS (↓) (*p* = n.s.) **INTERVENTION GROUP (pre-test *vs*. 3-mo FU)** CS (↓) (*p* = n.s.) BO (↑) (*p* = n.s.) STS (↓) (*p* = n.s.) **INTERVENTION GROUP (pre-test *vs*. 3-mo FU)** CS (↑) (*p* = n.s.) BO (↓) (*p* = n.s.) STS (↓) (*p* ≤ 0.05)	STS scores declined immediately after the program. Maintained at 3- and 6-month follow up. Improved Impact of Event scores showing a statistically significant improvement in CF resilience.
[36]	Professional nurses (*n* = 7) 85.7% female Age Range = 30–45 years	CF	Pre/Post—Transcranial Direct Current Stimulation (tDCS) Timed series counterbalanced research design. 18 sessions of tDCS over a 6 week period.	Compassion Fatigue Scale (CFS)	EAI Resilience Scale PSS	6 weeks post baseline	**INTERVENTION GROUP (pre- *vs*. post-test)** CFS (↓) (*p* = 0.46)	No effect on resilience, CF or stress. Lowered levels of resilience, CF and decreased empathy are significant predictors of BO.
[43]	Pediatric nurses (*n* = 80) Intervention Group (*n* = 42) Control Group (*n* = 38) 100% female IG Age: *M* = 49.3 CG Age: *M* = 47.7	BO, CS, CF	Quasi-random control trial—six, 1 h sessions once a week for 12 weeks. Sessions aimed to improve professional self-efficiency and included theoretical knowledge, experiential exercises, and homework tasks.	ProQoL, V5	DHSES Traumatic Event Questionnaire RSE Mastery Scale Hope Scale	12 weeks post baseline	**INTERVENTION GROUP (pre- *vs*. post-test)** CS (↑) (36.52 *vs*. 53.64) (*p* ≤ 0.001) BO (↓) (51.18 *vs*. 45.34) (*p* = n.s.) STS (↓) (51.46 *vs*. 46.78) (*p* = n.s.) **CONTROL GROUP (pre- *vs*. post-test)** CS (↑) (40.24 *vs*. 45.97) (*p* ≤ 0.001) BO (↓) (48.69 *vs*. 52.93) (*p* = n.s.) STS (↓) (48.38 *vs*. 51.33) (*p* = n.s.) **IG *vs*. CG** CS (*p* ≤ 0.001, ES ^b^ = 0.35) BO (*p* ≤ 0.001, ES ^b^ = 0.22) CF (*p* ≤ 0.001, ES ^b^ = 0.14)	Largest improvements were in the CS measure. Suggests focusing on trauma-related skills to reduce STS. This present-oriented, skill-focused intervention, that incorporates self-maintenance techniques as is future-oriented through development of positive outlook and hope, affects all aspects of STS – BO, CF and CF.
[37]	Staff nurses, nurse aides, secretaries, unit managers, supervisors (*n* = 74) Intervention Group (*n* = 46) Control Group (*n* = 28) Sex: Not reported Age: Not reported	CS, BO	Pre/Post—Mindfulness education and practice in 30 min classes once a week for 10 weeks.	ProQoL, V5	MAAS HCAHPS Individual and Work-Unit Level Stress	10 weeks post baseline	**INTERVENTION GROUP (pre- *vs*. post-test)** CS (↓) (53.20 *vs*. 52.93) (*p* = 0.76) BO (↓) (46.20 *vs*. 45.71) (*p* = 0.55) **CONTROL GROUP (pre- *vs*. post-test)** CS (↑) (53.77 *vs*. 54.25) (*p* = 0.58) BO (↓) (46.05 *vs*. 45.00) (*p* = 0.22)	BO scores improved in the IG, but both CS and BO scores improved in the CG. Hospital may benefit from incorporating mindfulness training to reduce stress among nursing staff. Possible the intervention period was too short to see change in these measures.
[38]	Medical center personnel (*n* = 32) Intervention Group (*n* = 16) Control Group (*n* = 16) 87.5% female Age: *M* = 44.2	CS, STS, BO	RCT—mindfulness meditation, yoga movements, relaxation through music seven 1 h sessions and one 2 h session once a week for 8 weeks.	ProQoL, V5	Stress Biomarkers (Salivary α-amylase) PSS DASS MBI FFMQ	8 weeks post baseline	**INTERVENTION GROUP (pre- *vs*. post-test)** CS (↑) (*p* = 0.0314) BO (↓) (*p* = n.s.) **CONTROL GROUP (pre- *vs*. post-test)** CS (↑) (*p* = n.s.) BO (↓) (*p* = n.s.)	Intervention period too short to detect an effect. Specific aspects of mindfulness may be associated with better control of the deleterious effects of work stress.
[39]	Emergency nurses (*n*= 73) 81% female Age: 57% aged 31–50 years	CS, BO, STS, CF	Pre/Post—4 h interactive group seminar followed by individual and group exercises e.g., guided imagery, and multimedia resources (printed handouts, DVD, guided imaging/music CD, website with CF, CS and resiliency educational resources and publications.	ProQoL, V5	None	3–4 weeks post baseline	**INTERVENTION GROUP (pre- *vs*. post-test)** CS (↑) (40.3 *vs*. 42.2) (*p* = 0.004) BO (↓) (23.9 *vs*. 20.0) (*p* ≤ 0.001) STS (↓) (23.5 *vs*. 21.4) (*p* = 0.001)	10% reported higher CS, 34% reported fewer BO symptoms, 19% reported fewer STS symptoms. Organizational prevention programs may help maximize caregivers level of CS and reduce the risks of developing CF. Short pre/post period does not indicated long term improvement.
[42]	Disability sector workers (*n* = 34) Group 1 (*n* = 8) Group 2 (*n* = 6) Group 3 (*n* = 20) 58.8% female Age: *M* = 42.9, *SD* = 9.6	CS, BO, STS	Pre/Post—Training focused group meets once weekly for 8 session, each of 2 h duration. **Group Work**: Core mindfulness practices e.g., mindful breathing, body scan meditation, and mindful stretching, walking and sitting. **Home Work**: 40 mins/day, 6 days/week of mindfulness practice.	ProQoL, V5	PHQ-9 FFMQ PSS DASS PANAS SWLS CBI	8 weeks post baseline	**INTERVENTION GROUP (pre- *vs*. post-test)** CS (↓) (50.87 *vs*. 50.39) (*p* = n.s.) BO (↓) (49.76 *vs*. 48.59) (*p* = n.s.) STS (↓) (52.98 *vs*. 49.68) (*p* = n.s.)	Participants reported enhanced awareness of the signs and sources of stress. Positive changes in self-care attitudes, behaviours and interactions with colleagues and clients.
[40]	Hospice workers (*n* = 68) Intervention Group (*n* = 34) Control Group (*n* = 34) 84% female IG Age: *M* = 46.5, *SD*,14.8 CG Age: *M* = 42.0, *SD*,12.0	CS, BO	RCT—Single session Group Music Intervention for Grief Resolution (GMR-GR) to allow: Expression of grief feelings; Connect socially with colleagues experiencing similar feelings; and Participate in a grief ritual to farewell patients who’d died.	Compassion Satisfaction and Fatigue (CSF) Test	HCGI WES	Immediately post baseline and 30 days post baseline	**BETWEEN GROUP DIFFERENCES (IG *vs*. CG)** BO (*p* = 0.98) CF (*p* = 0.91)	No significant differences in BO, CF. Single session format could be a limitation. Results not generalizable.
[41]	Military and civilian RNs, LPNs ^d^, and medics (*n* = 93) Sex: Not reported Age: Not reported	CS, BO, STS	Pre/Post—Care Provider Support Program (CPSP) training on resiliency, coping, and CF.	ProQoL, V5	WCQ CD-RISC	30 days post session	**A paired-samples *t*-test determined that CPSP** CS (↓) (39.64 *vs*. 39.18) (*p* = 0.62) BO (↓) (28.71 *vs*. 19.79) (*p* ≤ 0.001) STS (↑) (19.25 *vs*. 20.14) (*p* = 0.20)	Research required with larger samples at multisite locations. CPSP training decreases BO in military and civilian RNs, LPNs, medics. Decreased BO may lead to a decrease in overall CF.

^a^ BO = burnout, CS = compassion satisfaction, STS = secondary traumatic stress/secondary traumatization, CF = compassion fatigue; ^b^ ES = Effect Size; ^c^ TBS = Team Building Questionnaire; MBI = Maslach Burnout Inventory; EAI = Empathy Assessment Inventory; PSS = Perceived Stress Scale; DHSES = Disaster-Helper Self-Efficacy Scale; RSE = Rosenberg Self-Esteem Scale; HS = Hope Scale; MAAS = Mindful Attention Awareness Scale; HCAHPS = Hospital Consumer Assessment of Healthcare Providers and Systems; DASS = Depression Anxiety Stress Scale; FFMQ = Five Facet Mindfulness Questionnaire; PHQ-9 = Patient Health Questionnaire (9); PANAS = Positive and Negative Affect Schedule; SWLS = Satisfaction With Life Scale; CBI = Copenhagen Burnout Inventory; HCGI = Hospice Clinician Grief Inventory; WES = Work Environment Scale; WCQ = Ways of Coping Questionnaire; CD-RISC = Connor-Davidson Resilience Scale; ^d^ Licensed Practical Nurses.

## Appendix A. Search Strategy

Search parameters were created to identify studies that met the following criteria:

1. Published in the past 25 years (January 1990–December 2015)
2. Promoted the reduction of CF or related work and/or non-work risk factors:
  - Targeting a known risk factor or a protective factor for CF
  - See key words below for “CF and related concepts” OR
  - Targeting CF directly
3. Included a valid outcome measure of CF
4. Targeted an at-risk occupational group
  - See key words below for “At-risk occupations”
5. Used methodology that included quantitative longitudinal measurement such as a quasi-experimental or experimental design
  - See key words below for “Design”
6. Targeting employed individuals as the population of interest.
  - See key words below for “Employment type”
7. Major database search engines used:
  - EMBASE, CINHAL, PsychInfo, Web of Science, PubMed, Scopus, Google Scholar
8. Exclude the following papers:
9. Unpublished work, opinion pieces, grey literature, editorials, qualitative researchJournals searched by hand:
  - Journal of Traumatic Stress
  - Work and Stress
  - Occupational and Environmental Medicine
  - Oncology Nursing Forum
  - Nursing Administration Quarterly
  - Health and Social Work

**Table A1:** Keywords used in Search Strategy.

Keywords						
CF & Related Concepts	And	At-Risk Occupations	And	Design	And	Employment Type
“compassion fatigue” **OR** “compassion” **OR** “fatigue” **OR** “secondary trauma” **OR** “secondary traumatic stress” **OR** “vicarious trauma” **OR** “burnout”		“child protection” **OR** “child services” “community service/s” **OR** “social worker” **OR** “crisis care” **OR** “crisis accommodation” **OR** “aged care” **OR** “residential care” **OR** “respite” **OR** “adoption services” **OR** “disability” **OR** “welfare” **OR** “youth services” **OR** “pastoral care” **OR** ‘firefighters” **OR** “police” **OR** “ambulance” **OR** “emergency services” “psych *” **OR** “counsellor” **OR** “mental health services” **OR** “refugee services” **OR** “family violence” **OR** “sexual assault” **OR** “disaster relief” **OR**“army” **OR** “military” **OR** “defence force/s”		“review” **OR** “systematic review” **OR** “meta-analysis” **OR** “case control” **OR** “RCT” **OR** “quasi-experimental” **OR** “intervention” **OR** “evaluation” **OR** “QED” **OR** “cohort” **OR** “cross-sectional” **OR** “longitudinal” **OR** “qualitative” **OR** “descriptive”		“worker” **OR** “employee” **OR** “professional” **OR** “officer” **OR** “emp *”
